# Clinical isolates of ST131 *bla*OXA-244-positive *Escherichia coli*, Italy, December 2022 to July 2023

**DOI:** 10.2807/1560-7917.ES.2024.29.8.2400073

**Published:** 2024-02-22

**Authors:** Aurora Piazza, Marta Corbella, Vittoria Mattioni Marchetti, Cristina Merla, Irene Mileto, Angela Kuka, Greta Petazzoni, Stefano Gaiarsa, Roberta Migliavacca, Fausto Baldanti, Patrizia Cambieri

**Affiliations:** 1 Department of Clinical, Surgical, Diagnostic and Paediatric Sciences, University of Pavia, Pavia, Italy; 2 IRCCS Fondazione Policlinico San Matteo, Pavia, Italy; 3 Microbiology and Virology Unit, IRCCS Fondazione Policlinico San Matteo, Pavia, Italy; 4 Specialization School of Microbiology and Virology, University of Pavia, Italy

**Keywords:** OXA-244, ST131, *Escherichia coli*, high-risk clone, surveillance, WGS

## Abstract

The dissemination of carbapenemase-producing *Escherichia coli,* although still at low level, should be continuously monitored. OXA-244 is emerging in Europe, mainly in *E. coli*. In Italy, this carbapenemase was reported from an environmental river sample in 2019. We report clinical isolates of OXA-244-producing ST131 *E. coli* in four patients admitted to an acute care hospital in Pavia, Italy. The association of this difficult-to-detect determinant with a globally circulating high-risk clone, ST131 *E. coli,* is of clinical relevance.

OXA-244, a single-point mutation variant (Arg214Gly) of OXA-48, is a rarely reported carbapenemase with reduced activity against carbapenems, first described in 2011 in a *Klebsiella pneumoniae* clinical isolate in Spain [1,2]. To date, the OXA-244 variant has mainly been described in *Escherichia coli* and found in several countries across the European Union, Lebanon, Russia and the United Kingdom [3-6]. Although the origin of this variant is not yet certain, there are reports of not healthcare-related community and environmental spread.

The aim of the present report was to investigate the genetic diversity of the ST131 *E. coli* isolated at the Fondazione IRCCS Policlinico San Matteo (OSM) in Pavia, a 1,000-bed teaching hospital, using whole genome sequencing (WGS), with a focus on the possible presence of OXA-244-producing strains.

## Case description

During the 10-month period from November 2022 to September 2023, we collected and sequenced 30 carbapenem-resistant *E. coli* (CR-Ec) isolates. Ten of them, collected from 10 patients in the period December 2022 to July 2023, belonged to the high-risk clone ST131. Bacteria of this ST were isolated at OSM throughout most of the study period. The patients had a mean age of 58.3 years, ranging from 35 to 90 years, and eight of the 10 were male. Two patients were admitted to the pneumology unit, two to the infectious diseases unit, three to intensive care units (ICU), two to surgery wards, and one to general medicine. Among the 10 ST131 CR-Ec isolates, six were isolated from rectal swabs, two from nasopharyngeal aspirate, one from a urine sample and one from a blood culture.

## Antimicrobial profiles

Species identification and determination of antibiotic susceptibility profiles were carried out by MALDI-TOF MS (Bruker Daltonics, Bremen, Germany) using the software Bruker Biotyper 3.1 and Phoenix M50 BD (Becton Dickinson, Franklin Lakes, United States (US)) and interpreted according to the 2023 European Committee on Antimicrobial Susceptibility Testing (EUCAST) clinical breakpoints [9]. The presence of carbapenem resistance mechanism was confirmed with the NG-test Carba 5 immunochromatographic assay (NG Biotech Laboratories). All 10 isolates were resistant to ertapenem, cephalosporins, amoxicillin/clavulanate, ciprofloxacin and levofloxacin. Seven of the 10 isolates were resistant to gentamicin, and only two were resistant to trimethoprim/sulfamethoxazole. All 10 *E. coli* ST131 strains were susceptible to meropenem, and nine were resistant to imipenem and amikacin (Table 1).

**Table 1:** Antibiotic susceptibility profiles of ST131 *Escherichia coli* strains, Italy, December 2022–July 2023 (n = 10)

Strain	Isolation date	Specimen	Ward	AMC S≤0,25 R>0,5	P/T S≤8 R>8	FEP S≤1 R>4	CTX S≤2 R>2	CAZ S≤1 R>4	CZA S≤8 R>8	C/T S≤2 R>2	CFX S≤8 R>8	CRX S≤1 R>2	CIP S≤0.25 >0.5	LVX S≤0.5 R>1	ERT S≤0.5 R>0.5	IMP S≤2 R>4	MEM S≤2 R>8	AK S≤8 R>8	CN S≤2 R>2	SXT S≤2 R>4
32829	Dec 2022	Blood	Intensive care unit	>32/2 (R)	>32/4 (R)	>16 (R)	>4 (R)	>16 (R)	0,5/4 (S)	>2/4 (R)	>16 (R)	>4 (R)	>1 (R)	2 (R)	1 (R)	2 (S)	0,25 (S)	≤4 (S)	≤1 (S)	>8/152 (R)
7662	Dec 2022	Rectal swab	Infectious diseases	>32/2 (R)	>32/4 (R)	>16 (R)	>4 (R)	>16 (R)	2/4 (S)	>2/4 (R)	>16 (R)	>4 (R)	>1 (R)	>8 (R)	>2 (R)	4 (I)	2 (S)	≤4 (S)	≤1 (S)	≤1/19 (S)
7730	Jan 2023	Urine	Infectious diseases	>32/2 (R)	>16/4 (R)	>8 (R)	>4 (R)	>8 (R)	0,125 (S)	32/4 (R)	>8 (R)	>4 (R)	>1 (R)	>2 (R)	>1 (R)	4 (I)	4 (I)	16 (R)	>4 (R)	≤1/19 (S)
7944	Feb 2023	Nasopharyngeal aspirate	Pneumology	>32/2 (R)	>16/4 (R)	8 (R)	>4 (R)	8 (R)	0,064 (S)	4/4 (R)	>8 (R)	>4 (R)	>1 (R)	>2 (R)	>1 (R)	0,5 (S)	0,25 (S)	8 (S)	>4 (R)	≤1/19 (S)
8136	Mar 2023	Rectal swab	Intensive care unit	>32/2 (R)	>32/4 (R)	>16 (R)	>4 (R)	>16 (R)	0,5/4 (S)	>2/4 (R)	>16 (R)	>4 (R)	>1 (R)	>8 (R)	>2 (R)	≤0,25 (S)	2 (S)	≤4 (S)	≤1 (S)	>8/152 (R)
8171	Apr 2023	Rectal swab	General medicine	>32/2 (R)	>32/4 (R)	>16 (R)	>4 (R)	>16 (R)	1/4 (S)	>2/4 (R)	>16 (R)	>4 (R)	>1 (R)	>8 (R)	>2 (R)	1 (S)	2 (S)	≤4 (S)	2 (S)	≤1/19 (S)
8270	May 2023	Urine	Intensive care unit	>32/2 (R)	>32/4 (R)	>16 (R)	>4 (R)	>16 (R)	4/4 (S)	>2/4 (R)	>16 (R)	>4 (R)	>1 (R)	>8 (R)	>2 (R)	1 (S)	2 (S)	≤4 (S)	≤1 (S)	≤1/19 (S)
8345	May 2023	Rectal swab	Surgery	>32/2 (R)	>32/4 (R)	8 (R)	>4 (R)	>16 (R)	0,5/4 (S)	>2/4 (R)	>16 (R)	>4 (R)	>1 (R)	>8 (R)	>2 (R)	8 (R)	2 (S)	≤4 (S)	≤1 (S)	≤1/19 (S)
8638	Jul 2023	Nasopharyngeal aspirate	Pneumology	>32/2 (R)	>64/4 (R)	8 (R)	>4 (R)	8 (R)	0,5/4 (S)	>4/4 (R)	>8 (R)	>4 (R)	>1 (R)	>1 (R)	1 (R)	≤0,25 (S)	0,25 (S)	8 (S)	>4 (R)	≤1/19 (S)
8718	Jul 2023	Rectal swab	Surgery	>32/2 (R)	64/4 (R)	>8 (R)	>4 (R)	>16 (R)	0,5/4 (S)	2/4 (S)	>8 (R)	>4 (R)	>1 (R)	>1 (R)	>1 (R)	≤0,25 (S)	≤0,125 (S)	8 (S)	2 (S)	≤1/19 (S)

AK: amikacin; AMC: amoxicillin/clavulanate; CAZ: ceftazidime; CFX: cefuroxime; CFZ: cefazolin; CIP: ciprofloxacin; CN: gentamycin; CRX: ceftriaxone; C/T: ceftolozane/tazobactam; CTX: cefotaxime; CZA: ceftazidime/avibactam; ETP: ertapenem; FEP: cefepime; IMP: imipenem; LVX: levofloxacin; MEM: meropenem; P/T: piperacillin/tazobactam; SXT: trimethoprim/sulfamethoxazole.

Interpretation according to EUCAST breakpoints [7] are displayed in brackets.

## Genetic characterisation of OXA-244-producing ST131

We performed WGS on the Illumina MiSeq platform. The reads obtained were de novo assembled with Shovill [8]. We reconstructed the resistome, plasmidome and virulome of the isolates using ResFinder, PlasmidFinder and the Virulence Factors Database (VFDB) via ABRicate (github.com/tseemann/ABRicate), and determined the multilocus sequence typing (MLST) profiles according to the Achtman scheme on Enterobase [9].

Nine of the 10 strains harboured a *bla*CTX-M gene, of which six were the *bla*CTX-M-15 and three the *bla*CTX-M-27 variant. Among carbapenemases, the *bla*KPC-3 was detected in two strains from different wards, *bla*NDM-1 in one isolate, and four isolates from three wards were positive for *bla*OXA-244 (OXA-244-Ec). Table 2 adds information on genomic resistance determinants and plasmids.

**Table 2:** Metadata and genomic results of ST131 *Escherichia coli* isolates, Italy, December 2022–July 2023 (n = 10)

Strain	Isolation date	Specimen	Serotype	FimH	Clade	Resistome	Plasmidome
32829	Dec 2022	Blood	O16:H5	H41	A	*aadA5, bla*CTX-M-15, ***bla*OXA-244**, *dfrA17, mphA, sul1*	Col156, ColRNAI, IncFIA, IncFIB, IncFII
7662	Dec 2022	Rectal swab	O25b:H4	H30	C1-M27	*aph(3”)-Ib, aph(6)-Id, bla*CTX-M-27, *bla*KPC-3, sul2, *tetA*	Col(MG828), Col156, IncFIA, IncFIB, IncFII
7730	Jan 2023	Urine	O25b:H4	H30	C2	*aac(3)-IIe, aac(6')-Ib, bla*CTX-M-15, *bla*OXA-1, ***bla*OXA-244**	IncFIA, IncFIB, IncFIC
7944	Feb 2023	Nasopharyngeal aspirate	O25b:H4	H30	C2	*aac(3)-IIe, aac(6')-Ib, bla*CTX-M-15, *bla*OXA-1, ***bla*OXA-244**, *tetA*	Col156, IncFIA, IncFIB, IncFIC
8136	Mar 2023	Rectal swab	O25b:H4	H30	C1-M27	*aadA5, aph(3”)-Ib, aph(6)-Id, bla*CTX-M-27, *dfrA17*, *mphA, sul1, sul2, tetA*	Col(MG828), Col156, Col8282, ColRNAI, IncFIA, IncFIB, IncFII
8171	Apr 2023	Rectal swab	O25b:H4	H30	C2	*aph(3)-Ib, bla*CTX-M-15, *bla*TEM-235, *sul2*	Col(MG828), IncB/O/K/Z, IncFIB, IncFII
8270	May 2023	Urine	O25b:H4	H30	C1-M27	*bla*CTX-M-27	Col(BS512), Col(MG828), Col156, IncFIA, IncFIB
8345	May 2023	Rectal swab	O25b:H4	H30	C	*bla*KPC-3, *bla*SHV-158, *bla*TEM-1, *tetA*	Col(MG828), Col156, Col8282, IncFIA, IncFIB, IncFII, IncN, IncX3
8638	Jul 2023	Nasopharyngeal aspirate	O25b:H4	H30	C2	*aac(3)-IIe, aac(6’)-Ib, bla*CTX-M-15, *bla*OXA-1, ***bla*OXA-244**, *tetA*	IncFIA, IncFIB, IncFIC
8718	Jul 2023	Rectal swab	O25b:H4	H30	C2	*aac(6’)-Ib, aph(3’)-VI, bla*CTX-M-15, *bla*NDM-1, *bla*OXA-1, *dfrA5, mphA, mphE, msrE, qnrS1, sul1, sul2*	Col156, ColRNAI, IncFIB, IncFIB(pNDM-Mar), IncFII, IncHI1B, IncI1

Nine of the 10 ST131 strains showed the O25b:H4 serotype with a H30 fimbrial type, while the remaining, *bla*OXA-244-harbouring strain showed the O16:H5 with a H41 fimbrial type. Concerning the ST131 sublineages, three isolates belonged to C1-M27 (positive for *bla*CTX-M-27), five belonged to C2 and produced CTX-M-15, one belonged to clade C and encoded the carbapenemase KPC-3, and one to clade A with the H41 fimbrial type (Table 2).

**Figure 1:**
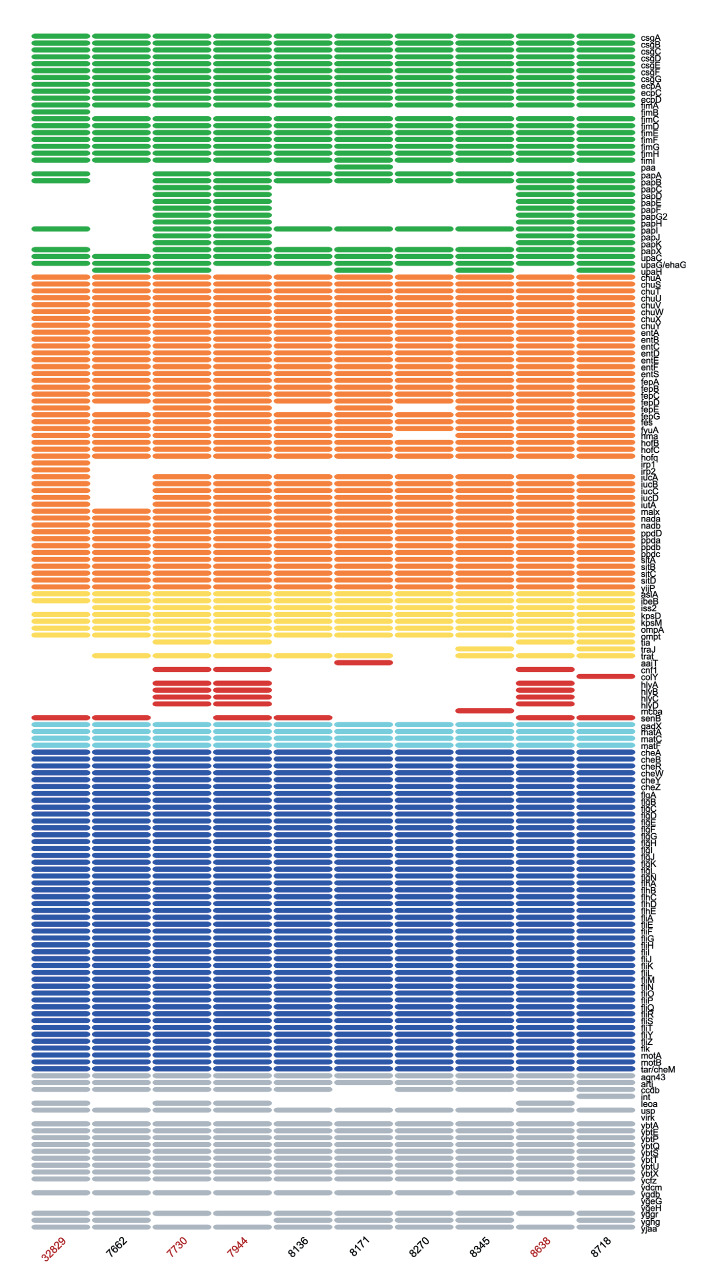
Heatmap representation of the virulence genes of *Escherichia coli* ST131 isolates, Italy, December 2022–July 2023 (n = 10)

All strains showed a conserved virulence pattern, associated with ST131 isolates, including genes (operons/gene groups) for iron uptake systems (*sitABCD, chuA, fyuA and malX*), for adhesion factors (*ecpACD* and *fimH*) and for invasion (*kpsM II* and *ibeB*). The operon *iucABCD* and the determinant *iutA* were identified in all but one strain. Among genes encoding for toxins, all 10 strains presented the *usp* gene, encoding a colicin-like toxin, six strains contained *senB,* the gene for enterotoxin TieB. The three OXA-244-positive O25b:H4 isolates carried in addition the cytotoxic necrotising factor 1 gene *cnf1,* and the hemolysin gene *hlyA* (Figure 1).

Green: adhesion; orange: metabolism; yellow: invasion; red: toxins; light blue: regulation; blue: chemotaxis; grey: others. The four OXA-244-producing *E. coli* ST131 are labelled in red.

The plasmid content showed that the IncF family with IncFI replicons was present in all 10 isolates, followed by IncFII which was found in six isolates. Among plasmids of Col-type, the Col156 plasmid was found in seven strains, co-existing in four cases with other specific Col-like replicons Col(MG828) and Col8282. Less frequent plasmid families such as IncX3, IncN and IncB/O/K/Z were also identified (Table 2).

**Figure 2:**
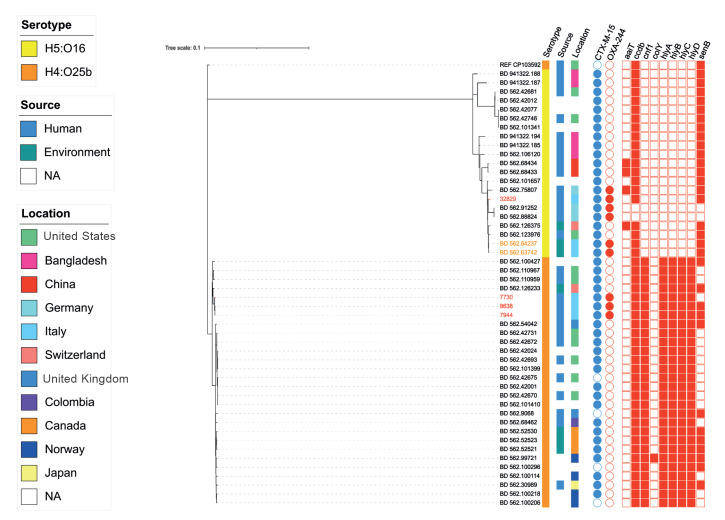
Phylogenetic analysis of OXA-244-producing *Escherichia coli* ST131, Italy, December 2022–July 2023 (n = 4)

The *bla*OXA-244 gene was found to be part of the Tn*1999.2 *transposon in all four strains; the association of *bla*OXA-244 with Tn1999.2 has already been described in the literature, usually with a chromosomal location [4,10]. Single nucleotide polymorphism (SNP)-based maximum-likelihood phylogeny was inferred using P-DOR [11] on the four OXA-244-Ec and the 46 most similar high-quality genomes belonging to ST131 available from the Bacterial and Viral Bioinformatics Resource Center (BV-BRC) database (https://www.bv-brc.org) (Figure 2). Based on the SNPs matrix, our four OXA-244-Ec belonged to two different clusters. The isolate 32829 was closely related to a small cluster of OXA-244-Ec collected from a rectal swab in Germany in 2019 [3]. Moreover, isolate 32829 revealed a relevant identity (SNP range: 194–197) with two other OXA-244-Ec collected from a water basin in Italy in 2019, in the same geographical area as OSM [10]. In contrast, the remaining three strains showed a monophyletic relation (SNP range: 52–95) and clustered together with clinical and environmental strains of CTX-M-15-producing *E. coli* ST131 collected in the US and in Switzerland, respectively. These data suggested a separate importation of OXA-244-Ec to the OSM.

NA: not available.

Maximum-likelihood phylogeny of 52 *E. coli* ST131 strains inferred on coreSNPs visualised using iTOL v6, including the four Italian OXA-244-producing *E. coli* ST131 (red labels) and 48 from the BV-BRC database (grey labels). Labelled in orange are the two Italian OXA-244-producing *E. coli* ST131 collected from water. The presence/absence matrix shows the pattern of resistance determinants (blue and red dots), toxin determinants (red square) and resistance determinants (red).

## Epidemiological investigation

The four *bla*OXA-244-positive *E. coli* were collected from four patients admitted to three different OSM wards at distinct times. The first isolate was collected in December 2022 from an ICU, the second in January 2023 on the infectious diseases ward, and the remaining two in the pneumology unit in February and July 2023. The two patients admitted to the pneumology ward did not overlap, with a gap of 5 months between their hospitalisations. The epidemiological investigation into possible contact among the patients revealed that three of them had been admitted to the ICU in February, October and November 2022, with no overlap. Environmental samples were not taken on the above wards, but no other similar strains have since been identified in any patient samples. The four patients had been transferred to OSM from other hospitals but were negative when screened for carbapenem-resistant *Enterobacterales *at admission to OSM. In 2019, an OXA-244-producing ST131 H5:O16 *E. coli* had been detected in canal water in the same geographical area [10], but we could not clearly assess the role of the environment or the community setting as a reservoir for such a strain.

## Discussion

*Escherichia coli *belonging to sequence type (ST) 131 is well known as a successful high-risk multidrug-resistant clone. Typically, isolates belonging to ST131 clade C (the most prevalent), are resistant to fluoroquinolones, due to mutations in the quinolone resistance-determining region, and to third-generation cephalosporins, due to the association with *bla*CTX-M-type determinants. However, they mostly remain susceptible to carbapenems [12]. In July 2021, a risk assessment published by European Centre for Disease Prevention and Control (ECDC) pointed out an increase in the prevalence of OXA-244-producing *E. coli* in Europe and evidence of healthcare-associated transmission [13,14], in contrast to what had been pointed out in the literature since early 2021. OXA-244 is known to be a difficult-to-detect carbapenemase, due to its weak hydrolytic activity towards carbapenems and temocillin and, thus, its difficulty to grow on selective and screening media [6]. To date, OXA-244-producing *E. coli* is predominantly found in ST38 but reported also in several other clonal groups such as ST10, ST69, ST167, ST361 and ST3268 [5,15]. The ability of OXA-244-producing *E. coli* ST131 to occur in clinical settings has already been reported in Germany and France, while in Italy, the sole report had been from water environment [3,6,10,15]. Here we describe the first evidence of such strains in a clinical setting in Italy. The lack of environmental sampling on the involved wards represents a limitation; indeed, the entry timeline of OXA-244-Ec in the OSM is hard to track. Nevertheless, the association of the difficult-to-detect determinant OXA-244 with a high-risk clone such as ST131 represents a concern for global health. 

## Conclusion

The presence of OXA-244 in the healthcare setting poses an additional challenge for microbiological diagnosis and surveillance. The source and route of transmission of OXA-244-producing *E. coli* in Europe is still unclear, and there is a need for further investigations in order to implement evidence-based control measures. It is important to not underestimate the sole ertapenem resistance, and to use rapid tests for the detection of carbapenemases in patients on wards such as ICUs, followed by molecular-based characterisation of the specific resistance genes, in order to rapidly initiate targeted and specific antibiotic therapy when required.
